# The brain shock index: repurposing the Lindegaard ratio for detecting cerebral hypoperfusion in children with cerebral malaria

**DOI:** 10.1186/s13089-025-00430-8

**Published:** 2025-05-30

**Authors:** Nicole F. O’Brien, Taty Tshimanga, Florette Yumsa Mangwangu, Ludovic Mayindombe, Robert Tandjeka Ekandji, Jean Pongo Mbaka, Tusekile Phiri, Sylvester June, Montfort Bernard Gushu, Hunter Wynkoop, Marlina Lovett

**Affiliations:** 1 Department of Pediatrics, Division of Critical Care Medicine, Nationwide Children’s Hospital, The Ohio State University, 700 Children’s Drive, Columbus, OH 43502 USA; 2 Departement de Pediatrie, Cliniques Universitaires de Kinshasa, Hopital Pediatrique de Kalembe Lembe, Kinshasa, Congo; 3 Universite De Kinshasa, Hopital Pediatrique de Kalembe Lembe, Kimwenza, Democratic Republic of Congo; 4 Universite des Sciences et des Technologie de Lodja (USTL), L’Hopital General de Reference de Lodja, Sankuru District Lodja, Republique Democratic du Congo, Lodja, Congo; 5 Queen Elizabeth Central Hospital, The Blantyre Malaria Project, Private Bag 360, Blantyre 3, Chichiri, Malawi

**Keywords:** Transcranial doppler ultrasound, TCD, Brain shock, Brain shock index, BSI, POCUS

## Abstract

**Background:**

Transcranial doppler ultrasound (TCD) allows for the assessment of the cerebrovascular hemodynamics in critically ill children. Given the increasing availability of machines equipped with TCD capabilities globally, it may be a useful approach to detect cerebral hypoperfusion and guide neurologic resuscitation for pediatric patients in resource limited settings where other neuromonitoring techniques are unavailable. However, the current need to evaluate waveform characteristics and to age correct values to determine if a study is abnormal decreases the feasibility of using point of care TCD in this way. The brain shock index (BSI), a repurposing of the Lindegaard Ratio, overcomes these limitations.

**Methods:**

We performed a prospective study of children with cerebral malaria (CM). On admission and daily thereafter, TCD was used to evaluate the middle cerebral (MCA) and extra-cranial carotid arteries (Ex-ICA), and the BSI was calculated bilaterally (MCA mean flow velocity ((Vm))/Ex-ICA Vm). Neurologic outcome at discharge was assessed.

**Results:**

A cohort of 291 children with CM were evaluated. BSI calculation was successful in all of them. The mean time to perform TCD and calculate the BSI was 4 ± 2 min. Overall, 222 participants (76%) had a good outcome and 69 (24%) a poor outcome. The BSI had an AUC of 0.98 (95% CI 0.97–0.99, *p* < 0.0001) to predict death or moderate to severe disability. The highest sensitivity and specificity of the BSI to predict adverse outcomes occurred at a cut off value ≤ 1.1. The adjusted odds ratio of poor outcome was 3.2 (95% CI 1.6–6.1, *p* = 0.001) if any BSI measurement during hospitalization fell below this threshold. No intracranial pressure monitoring was available to determine the relationship between the BSIs and an invasively measured cerebral perfusion pressure.

**Conclusion:**

The BSI is a rapid, feasible point of care ultrasound measurement of cerebral hypoperfusion, with values ≤ 1.1 strongly correlating with poor neurologic outcomes in children with CM. Future studies should be performed to assess the utility of BSI to detect the presence and measure the severity of reduced cerebral perfusion pressure in other populations of critically ill children.

## Background

Transcranial doppler ultrasound (TCD) can be used to assess the cerebrovascular hemodynamics of critically ill children. [1–8 In recent surveys of American and European pediatric intensivists, 26–40% of respondents used point of care neurosonography with TCD to determine when to obtain diagnostic imaging or to proceed with neurosurgical/neurovascular procedures, to guide the placement of intracranial pressure (ICP) monitors, to manipulate mechanical ventilation, and/or to determine cerebral perfusion pressure (CPP) goals. [9, 10] As TCD software is now available on multi-purpose ultrasound machines, it is also emerging as an invaluable resource to evaluate the cerebral vasculature of critically ill children in resource limited settings where other neuroimaging or neuromonitoring equipment is rarely available. [11– 15.

**Fig. 1:**
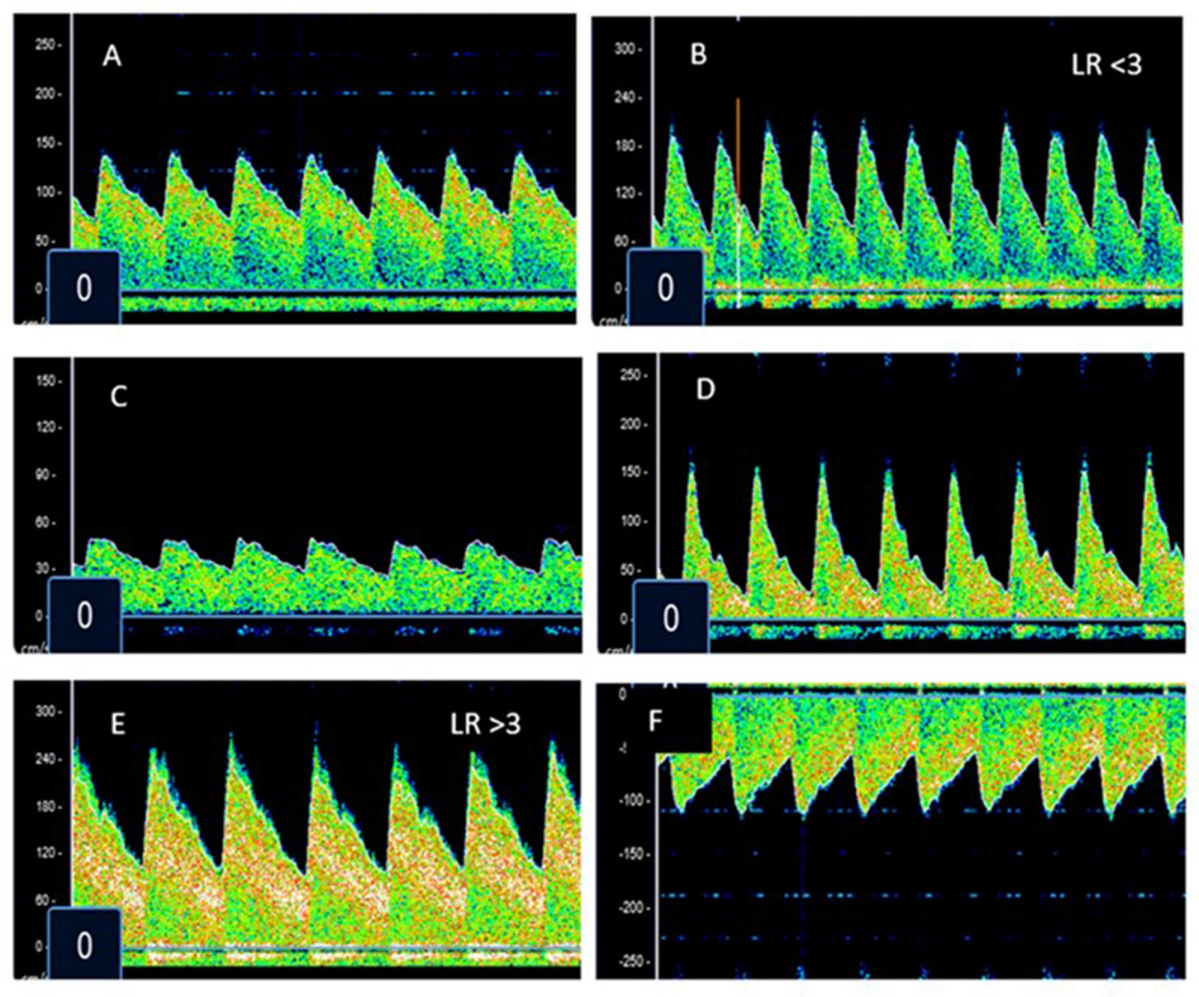
Previously described TCD phenotypes in pediatric cerebral malaria. **A** = normal, **B** = hyperemia, **C** = low flow, **D** = microvascular occlusion, **E** = vasospasm, **F** = isolated posterior high flow (IPH)

Malaria affects 249 million individuals and results in ~ 608,000 deaths annually, > 80% of which occur in African children.^16^ Cerebral malaria (CM) is a severe form of the disease that, even with effective antimalarial drugs, results in case fatality rates of 15–30%.[17–19] Among survivors, 30–50% are left with long-term neurologic complications including motor deficits, cognitive and behavioral issues, and epilepsy. [20–22] Diagnostic TCD has been used to evaluate the neurovasculature of children with the disease. Five different phenotypes of altered cerebral blood flow have been identified in CM (Fig. 1). [23, 24] Those classified as “low flow” experience the highest rates of death and disability at hospital discharge, likely due to secondary ischemic injury in the setting of impaired CPP.

To make the delivery of CPP targeted therapies to thousands of children with CM in resource limited environments feasible, an approach to determine critical reduction in cerebral perfusion that does not rely on invasive measurements of blood pressure or ICP nor on detailed interpretation of a full diagnostic TCD examination is necessary.[25] We therefore adapted the use of the TCD derived Lindegaard ratio (LR) and dubbed it the “*brain shock index (BSI*)”. The LR is calculated as: middle cerebral artery (MCA) mean flow velocity (Vm)/ extracranial internal carotid artery (Ex-ICA) Vm.^26^ The LR is validated to determine if *elevated* flow velocities in the anterior circulation are occurring due to hyperemia (where the LR is < 3) or cerebral vasospasm (where the LR is ≥ 3). The LR has never been used to evaluate low MCA flow.

Under the assumption that intracranial hypertension leading to decreased CPP would result in decreased MCA flow, but Ex-ICA flow would be unchanged, we hypothesized that lower BSI values would be associated with secondary brain injury exhibited as neurologic morbidity and mortality in a cohort of children with cerebral malaria. [27–29] As the BSI is a ratio, it is unnecessary to interpret waveforms or age correct flow velocities. We therefore also hypothesized that BSI determination would be feasible and rapidly performed in most children.

## Methods

### Patient population and general care of CM patients

This prospective, observational study occurred at Kalembe Lembe Children’s Hospital in the Democratic Republic of the Congo (DRC), Lodja District Referral Hospital in the DRC, and Queen Elizabeth Central Hospital/The Paediatrics Research Ward in Blantyre, Malawi from January 2021-July 2024. Parents or guardians of all children 6 months to 12 years of age who met the World Health Organization (WHO) case definition of cerebral malaria (*Plasmodium falciparum* parasitemia, Blantyre Coma Score ((BCS)) ≤ 2, and no other discernable cause of encephalopathy) were approached for enrollment. All parents provided written informed consent. Children with sickle cell disease (known or suspected) were excluded, given the high frequency of abnormal TCD examinations in this population. Likewise, given the unknown impact of severe malnutrition (mid-upper arm circumference < 11 cm) or advanced HIV disease (known HIV positive status with severe wasting) on TCD examinations, these children were also excluded.

Demographic data, vital signs, and physical examination findings were collected. All patients received intravenous artesunate according to the WHO guidelines. Patients received 20 mL/kg of whole blood if admission packed cell volume (PCV) was < 15% or > 15% but with signs of intolerance (defined as respiratory distress or hemodynamic compromise with a capillary refill time > 2 s, weak pulse, and/or cool extremities). Intravenous dextrose (1 mL/kg of dextrose 50%) was given when documented hypoglycemia occurred (< 3 mmol/L). Clinical seizure activity was treated with 0.2 mg/kg of diazepam followed by phenobarbital (20 mg/kg) if seizures persisted. Finger-prick samples were analyzed to determine parasite species and density, packed-cell volume (PCV), and blood glucose and lactate concentrations (Aviva Accu-Check, Zurich, Switzerland and Arkray Lactate Pro 2, Kyoto, Japan). An admission lumbar puncture in the lateral decubitus position was performed, opening pressure (OP) measured, and the cerebrospinal fluid was analyzed.

A bedside, point of care (POCUS) TCD was performed using a commercially available non-imaging unit (NovaSignal, Los Angeles, California, USA) within 4 h of presentation. The Vm in the right and left MCAs and Ex-ICAs were measured one time in the M1 segment of the vessel just distal from the takeoff of the anterior cerebral artery. The Vm in the MCA was divided by the Vm in the ipsilateral Ex-ICA for each individual to determine the *brain shock index (BSI)* (BSI = MCA Vm/Ex-ICA Vm). The right and left BSIs were both calculated and recorded. TCD was repeated daily through hospital day 6, normalization of the coma score, or death/discharge, whichever came first.

TCD studies were performed by individuals who had successfully completed the sub-Saharan African TCD Academy course content, which includes: (1) 12 online didactic lectures designed to teach the basic concepts of TCD science including neuroanatomy, scanning techniques, typical doppler waveform characteristics, diagnostic criteria for common pediatric disease conditions, and clinical applications in children; (2) a hands-on introduction to TCD training session to develop TCD scanning proficiency; (3) 100 case studies aimed at developing interpretation skills; and (4) a written examination. [30] The course was considered successfully finished when didactics and case studies were reviewed, the participant demonstrated proficiency in TCD scanning (each measurement of a complete TCD examination had a coefficient of variation < 10% from that of the trainer ((NF O’Brien)), and the written examination was passed.

## Outcomes

The Pediatric Cerebral Performance Category (PCPC) scoring system is a tool that was developed to measure and quantify morbidity after pediatric critical illness. [31, 32] Scores range from 1 to 6, with 1 being a normal functional level and 6 being death. Other values represent progressive impairment: 2 = mild disability (alert and able to interact at an age appropriate level but with mild cognitive, behavioral, or neurological deficits), 3 = moderate disability (alert and able to carry out age appropriate activities of daily life but with obvious cognitive or neurological deficits that limit function), 4 = severe disability (conscious but dependent on others for all daily functions), and 5 = vegetative state (any degree of coma or an inability to interact with the environment).

PCPC was scored at the time of hospital discharge by research staff. Children with a PCPC of 1 or 2 were considered to have a good outcome while those with a PCPC of 3 to 5 were considered to have survived with moderate to severe disability. Participants who died were scored as a 6. As neither invasive ICP measurements nor neuroimaging findings to grade cerebral edema were available for enrolled patients, PCPC scores were used as our primary outcome of interest.

## Statistics

Demographics, vital signs, clinical findings, and laboratory variables were summarized using frequencies with percentages and medians with interquartile ranges or means and standard deviations following assessment of normality. A receiver operating characteristic curve was generated to assess outcome prediction for BSI. Analysis revealed a BSI cut-off value with the highest sensitivity and specificity to predict outcomes. Differences in clinical and laboratory variables between children that had any BSI below versus all BSI measurements above this cut point were assessed using student’s t tests. Chi-squared tests were used to evaluate the differences in outcome between BSI groups. In all analyses, we considered a p-value of *≤* 0.05 to be a statistically significant difference between groups. All analysis were conducted using GraphPad Prism.

## Results

A total of 291 critically ill children with CM were prospectively enrolled in the study. No patient was excluded for an inability to find a TCD window. As CM results in global neurologic injury, < 5% difference between the right and left BSI was observed on all studies (*p* = 0.87). The mean time from study initiation to completion of the measurements of the bilateral MCAs and Ex-ICAs and calculation of the BSI was 4 ± 2 min.

Two hundred and forty-seven children (85%) were found to have a BSI > 1.1 on all study measurements whereas 44 (15%) had at least one point of care TCD with a BSI ≤ 1.1 during hospitalization. Those with any BSI ≤ 1.1 were significantly younger, had higher respiratory rate, lower glucose, and worse BCS at hospital admission (Table 1). Those children who had a BSI ≤ 1.1 at any point during their hospitalization had significantly worse outcomes than those that did not (Table 1, *p* = 0.001). The adjusted odds ratio of poor outcome was 3.2 (95% CI 1.6–6.1, *p* = 0.001) if any BSI measurement was ≤ 1.1.

**Table 1:** Admission demographics, vital signs, laboratory results, clinical features, and neurologic state at hospital discharge across the entire cohort as well as between those without and with brain shock index ≤1.1 on any measurement during hospitalization

Variable	Entire Cohort (*n* = 291)	BSI > 1.1 on all measurements (*n* = 247)	BSI ≤ 1.1 on any measurement (*n* = 44)	*p* value
*Demographics*				
Age (months), median [IQR]	51 [34, 78]	51 [36, 81]	40 [26, 68]	**0.01**
Male, n (%)	150 (52)	128 (52%)	23 (52%)	0.32
*Vital signs*				
Temperature (°C), mean (SD)	38.4 (± 1.1)	38.4 (± 1.1)	38.3 (± 1.1)	0.56
Heart rate (beats/min), mean (SD)	138 (± 25)	137 (± 24)	146 (± 28)	0.11
RR (breaths/min), mean (SD)	36 (± 11)	34 (± 10)	41 (± 15)	**0.009**
Oxygen saturation (%), mean (SD)	97 (± 3)	97 (± 3)	96 (± 3)	0.24
MBP (mmHg), mean (SD)	78 (± 12)	78 (± 12)	76 (± 12)	0.12
*Laboratory investigations*				
Packed Cell Volume (%), mean (SD)	26 (± 6)	25 (± 6)	27 (± 7)	0.06
Glucose (mmol/L), mean (SD)	5.8 (± 2.1)	6.0 (± 2)	5.3 (± 2)	0.03
Lactate (mmol/L), mean (SD)	5.1 (± 3.8)	5.0 (± 3.6)	5.7 (± 4.6)	0.48
*Pf*HRP2 (ng/mL), median [IQR]	591 [315,1365]	589 [312,1370]	593 [317,1363]	0.8
*Clinical Features*				
OP, median [IQR]	17 [12, 21]	17 [12, 21]	19 [14, 24]	0.22
MCA mean flow velocity, median [IQR]	94 [79,113]	102 [88,116]	82 [57,92]	**0.001**
Blantyre coma score, n (%)				**0.01**
0	33 (11%)	23 (10%)	10 (22%)	
1	104 (36%)	87 (35%)	17 (39%)	
2	154 (53%)	137 (55%)	17 (39%)	
*Outcome*				**0.001**
PCPC 1–2 (Good), n (%)	222 (76%)	198 (80%)	24 (55%)	
PCPC 3–6 (Poor), n (%)	69 (24%)	49 (20%)	20 (45%)	

n = number, SD = standard deviations, IQR = interquartile range, C = Celsius, min = minute, RR = respiratory rate, MBP = mean blood pressure, *Pf*HRP2 = Plasmodium falciparum histidine rich protein 2, OP = opening pressure, MCA = middle cerebral artery, PCPC = pediatric cerebral performance category

**Fig. 2:**
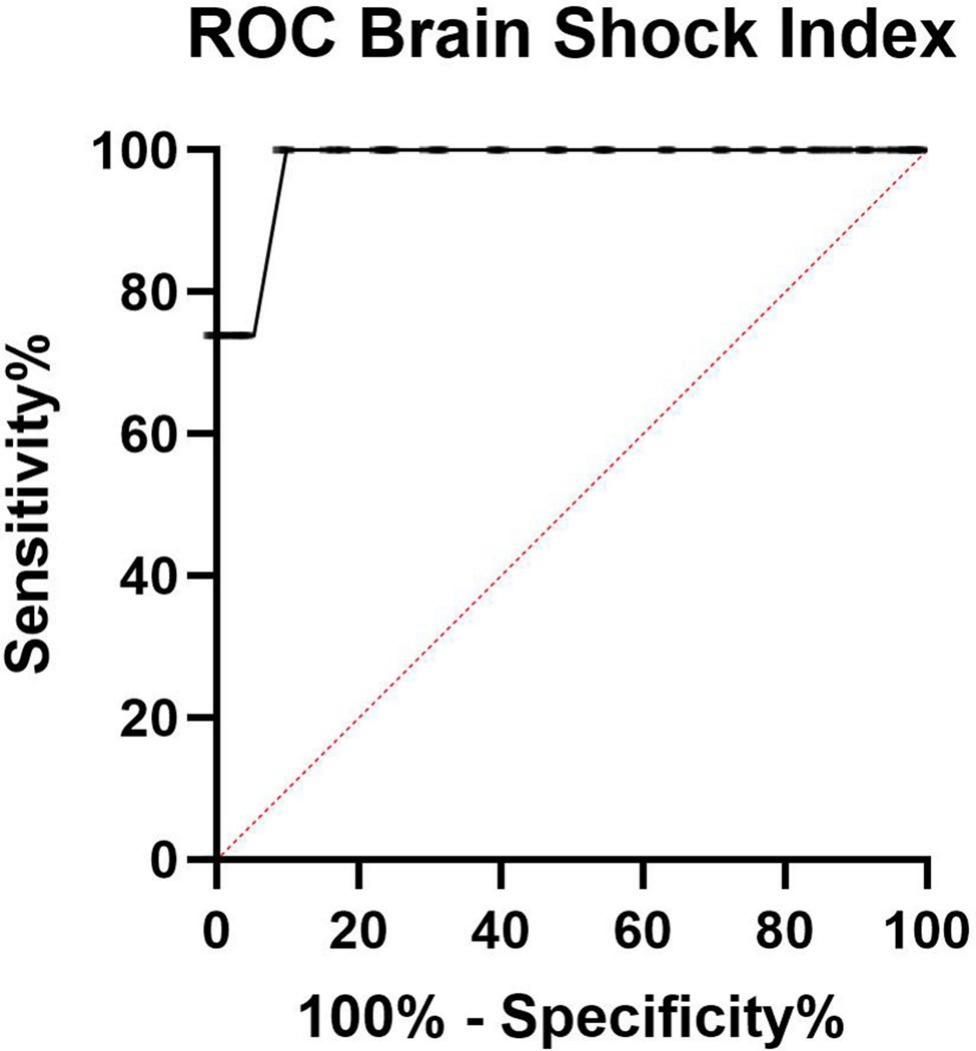
Receiver operator characteristic (ROC) curve demonstrating the predictive utility of the “brain shock index” for poor neurologic outcome in a cohort of children with cerebral malaria

**Fig. 3:**
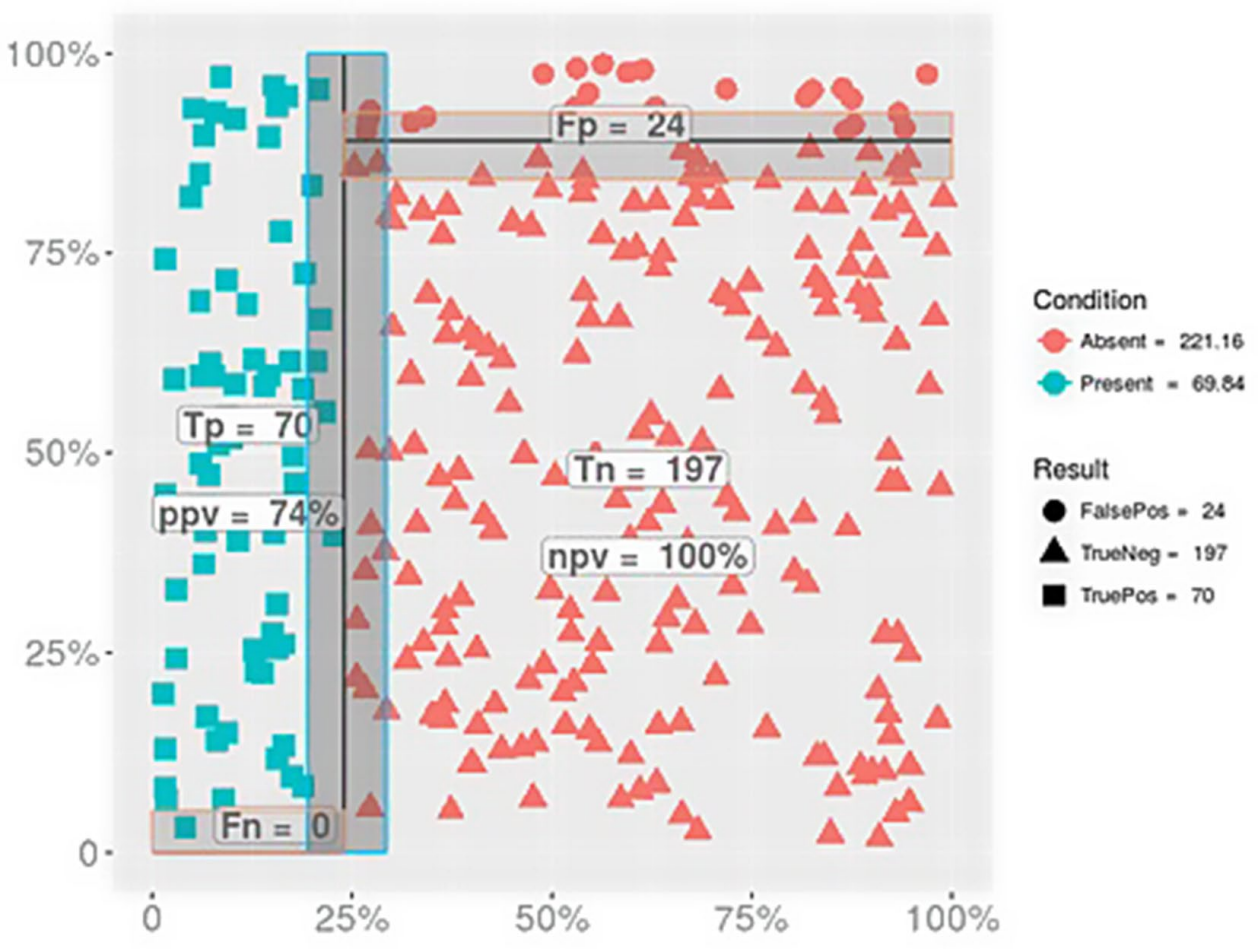
Positive and negative predictive value of the “brain shock index” for poor neurologic outcome in a cohort of children with cerebral malaria

Overall, 222 children (76%) had a good outcome and 69 (24%) a poor outcome. The BSI had an AUC of 0.98 (95% CI 0.97–0.99, *p* < 0.0001) to predict death or moderate to severe disability (Fig. 2). A BSI ≤ 1.1 had a 100% sensitivity (95% CI 98.6–100%), 89% specificity (95% CI 85–92%), 74% positive predictive value, and 100% negative predictive value for outcome prognosis (Fig. 3).^33^.

## Discussion

This study demonstrates that TCD can be used to quickly and easily determine the “brain shock index” that, with excellent sensitivity and specificity, predicts poor outcome in children with cerebral malaria. TCD was first used to evaluate the cerebrovascular hemodynamics of 50 Kenyan children with CM in 1996, with the hopes of gaining an improved understanding of the pathophysiologic contributors to brain injury in the disease [34]. Abnormal flows were reported in 30% of children, with perturbations associated with poor outcomes [30]. Our group has subsequently performed TCD on > 1200 children with the disease (with findings published for *n* = 616). [23, 24, 35] Based on well-defined alterations to TCD waveform morphology and measured flow velocities in all vessels of the Circle of Willis, five distinct phenotypes of deranged cerebral blood flow have consistently emerged (Fig. 1). The low flow phenotype is associated with the highest rate of death or neurodisability (RR 2.1, 95% CI 1.0-3.4). We have also found clear evidence of significant disruption of the cerebral energy metabolism in children with CM and the low flow phenotype. The median cerebrospinal fluid lactate: pyruvate ratio (LPR) in the low flow subset of children was 85 [IQR: 73–184], with values < 40 being considered normal [36]. Together, our studies show that TCD can be used as a “stethoscope into the brain” of children with CM to identify poor cerebral perfusion that leads to cerebral metabolic failure and worse outcomes.

However, as complete TCD studies evaluating all intracranial vessels, examinations performed for these prior reports require significant technical acquisition skills that limit the generalizability of its use as a neurodiagnostic tool in this setting. The BSI simplifies the approach by requiring assessment of only two easily visualized vessels (the MCA and Ex-ICA).

We also found the BSI advantageous to the traditional diagnostic TCD in these critically ill pediatric patients as it eliminated the need to interpret complex waveforms and to determine the number of standard deviations the measured flow velocities fell from age normative values. This allowed for rapid scanning and a BSI determination time of 4 min or less for most children in our cohort, making it feasible to use during emergent cerebral resuscitation events.

Further, use of the BSI may overcome an important obstacle the current TCD criteria for low flow has if this bedside tool were to be used in the future to guide therapeutic interventions for high risk children with CM. CM associated seizures are extremely common, prolonged, and refractory. [37] Thus, a majority of children presenting with CM will receive multiple doses of diazepam and/or phenobarbital prior to TCD study. Sedative agents such as these will reduce cerebral metabolic demand, and in doing so, reduce cerebral blood flow velocities by 21–47%[38]. This flow metabolism coupling is a normal physiologic response but, using our current diagnostic criteria, is still identified as a low flow phenotype. Distinguishing pathologic reductions in cerebral perfusion pressure that may benefit from therapeutic intervention from those with low flow that is simply physiologic is paramount. In pathologic alterations to the cerebrovascular hemodynamics, MCA flow will reduce, but Ex-ICA flow is maintained resulting in a “low” BSI. In flow-metabolism coupling, both the MCA and Ex-ICA flow will be low, resulting in a “normal” BSI. [27, 28] Future trials should confirm the ability of the BSI to make this important distinction.

The basis for measuring the BSI arises from the Monroe-Kellie doctrine. This doctrine states that the space of the cranial cavity is fixed so that the volume of its components (brain matter, blood, and cerebrospinal fluid) are interdependent. When cerebral edema occurs, the volume of the brain tissue increases and cerebral blood flow decreases. [39] MCA flows on TCD have been shown to negatively correlate with both the degree of cerebral edema on neuroimaging and with invasively measured ICP, whereas Ex-ICA flow is generally maintained. [27, 29, 40, 41] Therefore, when the ratio of flow between the MCA and Ex-ICA is low, a significant impairment in cerebral perfusion is present.

Given the pathophysiologic tenants of its measurement, we believe the BSI may have broader applicability as a screening and neuromonitoring tool in other critically ill pediatric patients with conditions other than CM. In particular, the BSI is most likely to be helpful in those at risk of or with diffuse brain injury and global cerebral edema from traumatic brain injury (abusive head trauma or diffuse axonal injury), hypoxic ischemic encephalopathy, neurologic infection, diabetic ketoacidosis with impaired consciousness, liver failure with hepatic encephalopathy, and for those undergoing extracorporeal support.

It remains less clear how the BSI would perform in those with focal neurologic illness or injury (traumatic brain injury (TBI) with focal intracranial hemorrhages, stroke, arteriovenous malformation rupture). In this setting, it may be possible that the BSI ipsilateral to the impacted side or significant side to side variability in the measured BSIs would be the most clinically meaningful. It is unlikely that the BSI would be helpful when impaired cerebral perfusion has a cardiovascular cause such as septic or cardiogenic shock as the Ex-ICA flow would be low in these situations and the measured BSI would be “normal”.

Last, while frequently disrupted in children with various forms of acute neurologic illness or injury, intact autoregulation may blunt the reductions in BSI as ICP increases and CPP decreases to a certain degree, resulting in a change to the clinically relevant threshold. [5, 6, 42] Future studies from other settings that include patients with heterogeneous diagnoses and those with both global and diffuse brain injury and various degrees of impairment to autoregulation should be done to explore if the determination of the BSI is a useful adjunct to clinical care in these situations.

There are limitations to our work. Participants were enrolled only when a TCD technician was available to perform the admission study, so the data presented here are not from consecutive patients. Also, based on operator availability, some patients had one or two TCD examinations missed. If all TCD studies had been performed, the values describing the relationship between the BSI and outcome may have differed slightly from what we report.

Another limitation is that invasive ICP monitoring was not available at any of the sites to allow us to determine if the BSI correlates directly with measured ICP/CPP. In future trials, if a strong relationship is identified, the BSI may not only be used as a prognostic biomarker but also to determine if/when invasive monitoring is indicated or to guide therapies to ensure ICP/CPP goals are met without monitoring.

The final limitation of the study is that no therapeutic interventions were undertaken for those with a BSI ≤ 1.1. It therefore remains unclear if treatment of a critically low BSI would improve rates of neurologic morbidity or mortality or if these were the moribund patients in the cohort. Future studies can also explore if treatment improves the BSI and/or has an impact on short and long-term outcomes.

## Conclusions

The point of care TCD derived brain shock index is rapidly calculated at the bedside, with levels ≤ 1.1 predicting poor outcomes in children with cerebral malaria. Future work should evaluate the utility of the BSI to be used as a screening and neuromonitoring tool in critically ill children at risk of neurologic injury with alternate diagnosis.

## Data Availability

The datasets used and/or analyzed during the current study are available from the corresponding author on reasonable request.
